# Novel insights into clear cell renal cell carcinoma prognosis by comprehensive characterization of aberrant alternative splicing signature: a study based on large-scale sequencing data

**DOI:** 10.1080/21655979.2021.1906096

**Published:** 2021-03-30

**Authors:** Dong Zhang, Wenjie Zhang, Rui Sun, Zhongxian Huang

**Affiliations:** a Department of Breast Surgery, Qilu Hospital, Shandong University, Jinan, China; b Cheeloo College of Medicine, Shandong University, Jinan, China; c Department of General Surgery, Shandong Provincial Qianfoshan Hospital, Shandong University, Jinan, China; d Department of Obstetrics and Gynecology, Qilu Hospital, Shandong University, Jinan, China; e Department of Urology, Jinan Central Hospital, Cheeloo College of Medicine, Shandong University, Jinan, China

**Keywords:** Clear cell renal cell carcinoma, alternative splicing, molecular subtype, prognosis, splicing factors

## Abstract

Clear cell renal cell carcinoma (ccRCC) is the most common type with poor prognosis in kidney tumor. Growing evidence has indicated that aberrant alternative splicing (AS) events are efficacious signatures for tumor prognosis prediction and therapeutic targets. However, the detailed roles of AS events in ccRCC are largely unknown. In our study, level 3 RNA-seq data was acquired from The Cancer Genome Atlas dataset and corresponding AS profiles were detected with the assistance of SpliceSeq software. A total of 2100 aberrant survival-associated AS events were identified via differential expression and univariate cox regression analysis. The final prognostic panel formed by 17 specific events was developed by stepwise least absolute shrinkage and selection operator (LASSO) penalty, with the area under curve (AUC) values of receiver operator characteristic (ROC) curves keeping above 0.7 spanning 1 year to 5 years. And the results from functional enrichment analyses are unanimous that autophagy could be a potential mechanism of splicing regulation in ccRCC. Furthermore, splicing regulatory network was constructed via Spearman correlation between splicing factors and AS events. Finally, unsupervised clustering analysis revealed three clusters with distinct survival patterns, and associated with specific clinicopathological phenotypes. In overall, we developed a robust and individualized predictive model based on large-scale sequencing data. The identified AS events and splicing network may be valuable in deciphering the crucial posttranscriptional mechanisms on tumorigenesis of ccRCC.

## Introduction

Renal cell carcinoma (RCC), originated from the renal epithelium, is the most common form of renal cancer and accounts for >90% of cancers in the kidney. Annually, over 290,000 new cases are diagnosed and approximate 134,000 deaths are recorded worldwide [1]. The disease encompasses several subtypes, of which clear cell RCC (ccRCC) is the most prevalent and contributes to the most cancer-related deaths [2]. Partial or radical nephrectomy is one of the effective methods to treat ccRCC. However, 20–30% of patients treated with surgery will relapse, despite having no evidence of metastases when diagnosed with ccRCC [3,4]. Similar to other solid tumors, ccRCC development and progression are characterized by aberrant genetic and protein expression. In the past decade, genetic alterations of ccRCC have been extensively investigated, revealing that specific genetic and epigenetic alterations are associated with carcinogenesis, recurrence and metastasis of tumor [1,5,6]. Hence, it is crucial to explore molecular mechanisms more deeply and excavate vital biomarkers that can improve the prognosis of ccRCC.

Protein diversity is fundamental for generating remarkable regulatory and functional complexity of human cells. A basic mechanism for protein diversity is the alternative modification and processing of pre-mRNA [7]. Alternative splicing (AS) is a post-transcriptional process which occurs in >90% human genes, engendering an enormous number of new isoforms from few sets of genes by inclusion or exclusion of different exons or parts of exons in pre-mRNA. This process has a significant impact on the diversity of both transcriptome and proteome of human [8,9]. AS plays a vital role in increasing the complexity of functional proteins and regulating of cell metabolism and cell-specific functions, which provides opportunity for tumorigenesis and progression [10]. Over the last two decades, molecular tools have been developed to correct or redirect AS events. It has been observed that a switch on particular AS event could occur in cancer-related genes [11]. Aberrant AS can lead to loss-of-function in tumor suppressors or activation of oncogenes [12]. Moreover, researches show that splicing factors (SFs) can not only regulate AS events directly, but also can be regulated by various signaling pathways, making AS sensitive to tumor microenvironment [13,14]. Accordingly, deciphering valuable AS events may help to elucidate underlying mechanisms of oncogenesis in ccRCC and provide new therapeutic approaches.

In recent years, even though the cancer-specific splicing variant has been widely detected and recognized in ccRCC tissues with the advancement of high throughput bio-technology [15,16], there is still limited understanding in its potential to be diagnostic, prognostic and predictive biomarkers as well as therapeutic targets. The rapid accumulation of sequencing resources, especially RNA-seq data, enabled us to quantitatively measure gene isoforms with better resolution and deeper coverage. However, to the best of our knowledge, there is a scarcity of studies integrating the large-scale high-throughput data and clinical information to illuminate the clinical significance of detailed splicing pattern at individual exon resolution, especially in ccRCC.

To comprehensively dive into clinically relevant splicing variants that may affect the major biogenesis, progression and molecular classification of ccRCC, we systematically profiled the genome-wide AS landscape based on a relatively large-scale sequencing cohort and identified ccRCC-related AS events. We further uncovered the potential biological functions of these events and their splicing regulatory relationships with key SFs. Moreover, integration of clinicopathological characteristics and RNA-Seq data provided insights into prognostic value and clinical significance of AS events. Finally, we discerned distinct splicing clusters of ccRCC with different survival patterns, and associated with different molecular subtypes. Our analysis depicts a complex splicing landscape, with several AS events revealed to be most valuable in deciphering the underlying mechanisms in the oncogenesis of ccRCC and serving as candidates of therapeutic targets to further validations.

## Materials and methods

### Data acquisition and pre-processing

Level 3 RNA-seq data and the corresponding clinical datasheets for ccRCC samples were downloaded from GDC data portal of TCGA website (https://portal.gdc.cancer.gov/) [17,18]. Analysis of AS profiles was performed with assistance of SpliceSeq software and TCGA SpliceSeq database, which evaluated the mRNA splicing pattern and quantified AS events with the Percent Spliced In (PSI) score calculated, ranging between 0 and 1 [19,20]. To generate a reliable set of AS events, we implemented the following filter criteria: percentage of samples with PSI score ≥75, PSI range across samples ≥0.05 and standard deviation of PSI score ≥0.02. And the remaining AS events with not available (Null) were imputed via the k-Nearest Neighbor (kNN) imputation algorithm [21]. Besides, our subsequent analyses were based on ccRCC cohort comprised of patients that meet the inclusion criteria [1]: a histological diagnosis of ccRCC [2]; patients who did not receive prior treatment, especially neoadjuvant chemotherapy [3]; patients with relatively complete clinical features including gender, age, tumor site, Fuhrman grade, pathological stage, T stage, M stage and overall survival (OS) information [4]; the follow-up time was no less than 30 days [5]; patients with corresponding mRNA splicing profiles. The present study fully satisfies the TCGA publication guidelines.

## Differentially spliced AS events analysis

To identify differentially expressed AS events within each AS type between ccRCC and normal tissues, student’s t-test, followed by an adjustment of *p*-value using the Benjamini-Hochberg correlation for multiple comparison, was applied. The |log2FC| > 1 and adjusted *p*-value < 0.05 indicated the AS events were significant differences between tumor and adjacent normal tissues.

## Identification of survival-associated AS events and functional enrichment analyses

After rigorous screening, a total of 455 ccRCC patients with differentially expressed AS profiles and survival information were subjected to subsequent analyses. For each specific AS event, patients were divided into two group based on median cut. A univariate Cox proportional hazard regression analysis was used to define the prognostic value of each AS event. In this analysis, AS events were regarded as significant at *p*-value < 0.05. And Upset plot, generated by UpsetR, was visualized to quantitatively analyze the gene interactions among the seven types of prognostic AS events [22]. Then to further shed light on the potential modifying mechanism of aberrant AS events on corresponding protein, the parent genes of these survival-associated AS events were sent for functional enrichment analyses via clusterProfiler R package, including Gene Ontology (GO) and Kyoto Encyclopedia of Genes and Genomes (KEGG) analysis [23]. The GO terms and KEGG pathways were considered significant with a threshold of false discovery rate (FDR) < 0.05.

## Dimension reduction and generation of AS signature

For avoiding multicollinearity of highly correlated variables, the Cox regression model, with least absolute shrinkage and selection operator (LASSO) penalty, was implemented to reduce dimension [24]. Firstly, among the top 20 most significant AS events in univariate analysis within seven AS types, the key prognostic biomarkers were selected to further develop prognostic signatures via LASSO-penalized method. Then we utilized the regression coefficients derived from fitting multivariate Cox model to multiply the PSI scores of prognostic indicators for constructing AS signature in each AS type. Furthermore, the candidate AS events from seven signatures were combined together to build the final AS panel for ccRCC cohort. Then we divided patients into low-risk and high-risk subgroups based on median value of each model, the Kaplan-Meier survival analysis and log-rank test were applied to verify the prognostic ability of prognostic models. Moreover, the predictive efficiency of each model was assessed by time-dependent receiver operator characteristic (ROC) curves. Therein, the LASSO Cox model was constructed with the ‘glmnet’ R package, using the 10-fold cross-validation and ‘lambda.min’ criteria such that the tuning parameters (lambda) were at the minimum [25]. The area under curve (AUC) of time-dependent ROC curve was calculated with ‘timeROC’ R package [26].

## Independence of final AS panel from clinicopathological features

To investigate the independent prognostic value of final AS panel from conventional clinicopathological characteristics (including age, gender, Fuhrman grade, pathological stage, T stage, M stage) in ccRCC patients, univariate followed by multivariate Cox regression analyses were conducted. Then to further confirm whether the final AS panel was of high applicability and robust in various subgroups, stratification Cox analyses were also performed.

## Construction and evaluation of the nomogram

In order to provide surgeons with a clinically relevant quantitative approach for predicting the short-term and long-term survival probability of ccRCC patients individually, we assembled a nomogram that integrated the final AS panel and independent clinicopathological risk factors based on the above results of multivariate analysis. Then the calibration curves were graphically assessed to determine whether the derived nomogram performed well compared to ideal model. Therein, the nomogram and calibration plot were depicted using rms package [27,28]. And Harrell’s concordance index (C-index) was calculated to estimate the discrimination ability of the nomogram with the help of Hmisc package. Moreover, the clinical usefulness of the nomogram, the AS panel and clinical risk factors were compared with the aid of decision curve analysis (DCA) [29–32].

## Gene set enrichment analysis (GSEA) of final AS panel

To further identify crucial biological processes and cancer-specific pathways related to the final AS panel, we performed a GSEA using the adjusted expression profiles for all transcripts. The annotated gene set files of ‘c2.cp.v6.2.symbols’ and ‘c5.bp.v6.2.symbols’ download from the ‘Molecular Signatures Databases (http://software.broadinstitute.org/gsea/msigdb)’ were employed for running GSEA using the java software (http://software.broadinstitute.org/gsea/downloads.jsp) [33,34]. Enrichment *p*-values were based on 1000 permutations and the significance threshold was set at nominal *p*-value < 0.05.

## Construction of splicing regulatory network

A list of 88 human SFs was created by integrating SpliceAid 2 database (www.introni.it/spliceaid.html) and the work from Xiong et al, which collected the experimentally validated SFs through hand-curated screening of literature and databases [35,36]. First, we compared ccRCC samples and adjacent normal samples to identify differentially expressed SFs using Student’s t-test. Then we assessed the correlation between the normalized expression value (variance stabilizing transformation via DESeq2 package) of SFs and OS through fitting univariate regression analysis in the entire cohort, where the SFs with *p*-value < 0.05 were selected as prognostic ones for further analysis [37]. The ‘surv_cutpoint’ function of the ‘survminer’ R package was used to iteratively determine the optimal cut points of prognostic SFs achieving the maximally selected rank statistics. Then Spearman correlation analyses were performed between expression value of prognostic SFs and PSI scores of the most significant AS events in each AS type. *P*-values were adjusted by Benjamini-Hochberg (BH) procedure and the significance threshold was set at an adjusted *p*-value < 0.05.

## Molecular subtyping for ccRCC based on aberrant prognostic AS profiles

AS events, especially the aberrant and survival-associated ones, may convey important information for identifying distinct molecular subtypes in ccRCC patients. Thus, the unsupervised cluster algorithm was implemented by ConsensusClusterPlus (R package) to identify robust molecular classification of ccRCC. The Elbow method and the Silhouette coefficient were combined to obtain the optimal number of clusters. Kaplan-Meier analysis was performed to validate the clinical outcomes of different molecular subtypes. The Chi-squared test and logistic regression analyses were applied to assess the associations between clinicopathological characteristics and the ccRCC subtypes that we identified.

## Statistical analysis

All statistical analyses were conducted using R language (version 3.5.2, https://www.r-project.org/) and considered two-sided *p*-values < 0.05 as statistical significance. Shapiro-Wilk test was utilized to test the normality of variables. For comparisons between two groups, unpaired Student’s t-test was used to estimate the statistical significance of normally distributed variables, whereas Mann-Whitney U-test was used to analyze the variables of skew distribution. Spearman correlation analysis was performed to estimate the coefficients and correlations between variables, and Chi-squared tests were used to analyze contingency tables. The R package survival was used to perform the univariate and multivariate Cox proportional hazard regression analysis. The LASSO-penalized Cox model was implemented to reduce dimension and select most significant biomarkers via R package glmnet. The sensitivity and specificity of prognostic models were evaluated with time-dependent ROC curves, quantified by AUC values using timeROC R package. The calibration, discrimination and clinical usefulness of prognostic model were measured by calibration plot, Harrell’s C-index and DCA, respectively. Additionally, consensus cluster, an unsupervised cluster algorithm, was implemented to do molecular subtyping for ccRCC samples via ConsensuClusterPlus R package.

## Results

Given the evidence that AS has emerging clinical potential in cancer diagnosis and therapy, a systematic analysis to delineate the landscape of splicing signature in ccRCC is lacking and greatly needed. Through our in silico analysis and screening, genome-wide AS events were profiled in ccRCC, with aberrant and survival-associated splicing variants identified. Further enrichment analyses confirmed parent genes of these AS events function in modifying ccRCC-related biological pathways. Some splicing events, delivered via machine-learning selection procedure, may be most valuable in elucidating the oncogenic properties of ccRCC, and provide clues of potential therapeutic targets to further validation. Additionally, consensus cluster identified AS clusters associated with prognosis and molecular subtypes.

## Overview of integrated AS events profiling in TCGA ccRCC cohort

**Figure 1.**
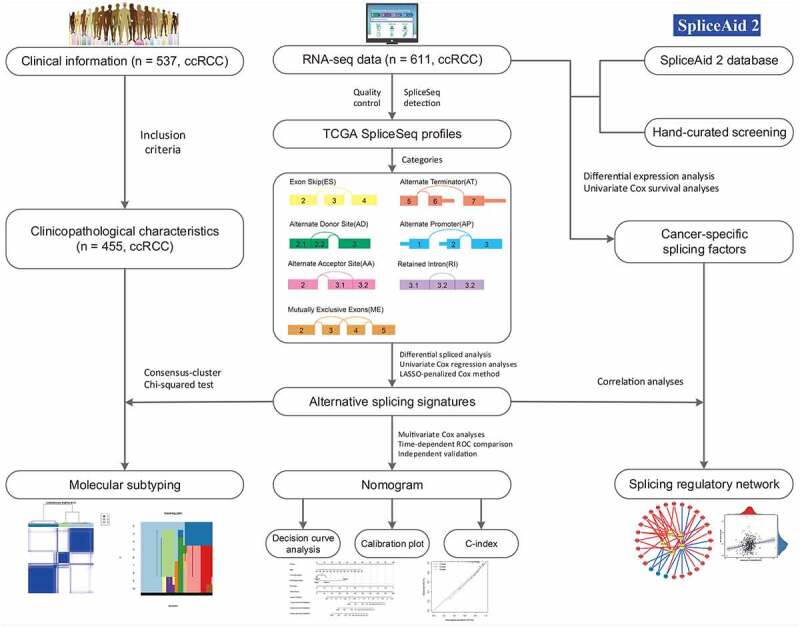
The flowchart for profiling the alternative splicing signatures in ccRCC patients in a relatively large-scale sequencing cohort

After a series of stringent filtering and screening, a total of 455 ccRCC patients from the TCGA with integrated mRNA splicing variants profiling and counterpart clinical information were retrospectively analyzed in depth. The included cohort was comprised of 155 female (34.1%) and 300 male (65.9%) patients, among which 145 (31.9%) died and the median survival time was 79.5 months. The median follow-up time of these patients with ccRCC was 37.0 months (range, 1.3–112.6 months). Detailed information of this study design are illustrated in Figure 1 as a workflow chart. For the entire cohort, the 1-, 3-, 5-year OS rates were 89.9%, 74.7% and 61.0%, respectively. Besides, we detected and quantifies the AS events of transcripts by using SpliceSeq software. According to the exclusive and distinct splicing pattern, these AS events can be roughly divided into seven particular types, including Exon Skip (ES), Alternate Terminator (AT), Alternate Promoter (AP), Retained Intron (RI), Mutually Exclusive Exons (ME), Alternate Donor site (AD) and Alternate Acceptor site (AA). As results, we obtained 32,487 mRNA splicing events from 9286 parent genes, which contains 2634 AAs in 1964 genes, 2366 ADs in 1751 genes, 6155 ATs in 2806 genes, 173 MEs in 167 genes, 11,371 ESs in 5230 genes, 2031 RIs in 1404 genes and 7757 APs in 3259 genes. And ES (35%), as the predominant type, accounts for more than one-third of all events, followed by AP and AT type in ccRCC and normal adjacent cancer tissues. We also noticed that one gene might possess two or more events. Considering this, Upset plot was generated to quantitatively visualize the intersecting sets among these seven AS types (Supplementary Fig. S1). Intriguingly, the vast majority of splicing variants were from the same genes while one gene could have up to five types of AS events, revealing the fact that AS hold largest potential on expanding transcript diversity and increasing proteome complexity.

## Identification of aberrant cancer-specific AS events in ccRCC

**Figure 2.**
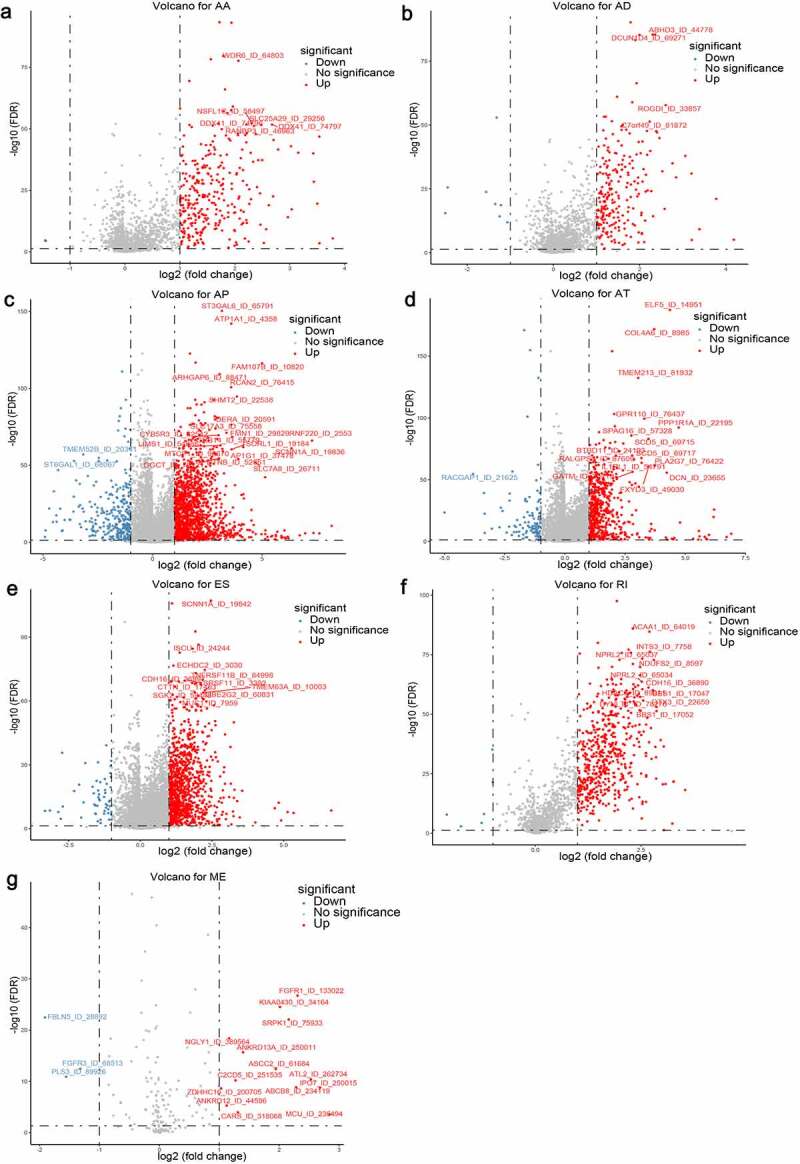
Volcano plots depicting differentially spliced events via Student’s t-test in seven alternative splicing (AS) types, including (a) Alternate Acceptor (AA), (b) Alternate Donor (AD), (c) Alternate Promoter (AP), (d) Alternate Terminator (AT), (e) Exon Skip (ES), (f) Retained Intron (RI) and (g) Mutually Exclusive Exons (ME). The *x*-axis represents the log2 transformation of fold change that the average PSI score in clear cell renal cell carcinoma (ccRCC) samples versus average score in normal tissues for each AS event, while the *y*-axis represents the negative log10 transformation of false discovery rate (FDR) for each comparison. The red and blue dots indicate the differentially expressed AS events with statistical significance (FDR < 0.05, |log2FC| ≥1). The assigned names of top ranked splicing events are shown in the plots

Considering the significantly spliced AS events between tumor and adjacent normal samples could play crucial roles in the tumorigenesis and development of ccRCC, we performed differential expression analyses with 72 normal tissues and 533 TCGA ccRCC tissues summarized. Using the preliminarily screening criteria mentioned above, we derived a list of 4660 aberrant splicing events, including 1069 ESs in 823 genes, 543 RIs in 438 genes, 1777 APs in 1470 genes, 719 ATs in 676 genes, 231 ADs in 204 genes, 304 AAs in 280 genes and 17 MEs in 17 genes. Interestingly, ES only contributes about 22.9% aberrant events even though it constitutes the highest proportion of AS events. As shown in Figure 2, most differential expressed AS events tend to be up-regulated in ccRCC tissues, suggesting the dysregulated splicing variants prefer to generate oncogenic transcripts rather than silence tumor suppressor transcripts.

## Construction of AS panel and evaluation of its predictive ability in ccRCC cohort

**Figure 3.**
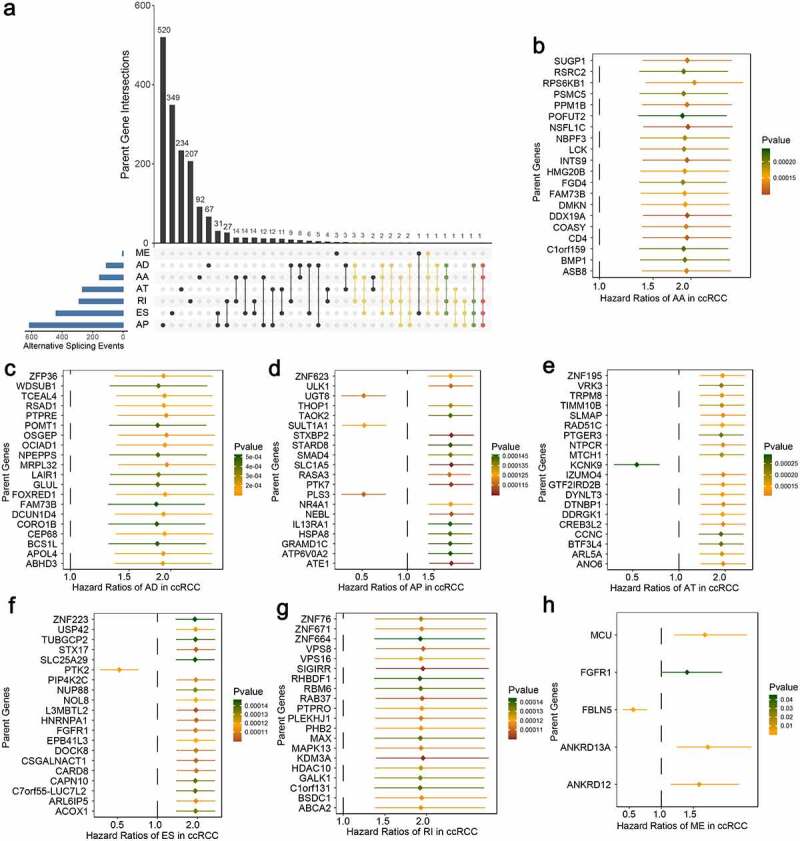
Univariate Cox regression analyses to identify aberrant survival-associated and the most significant splicing variants, which are candidates for further building predictive models in ccRCC. (a) Upset plot in ccRCC, showing the parent gene intersection among the seven types of AS events that simultaneously identified as cancer-specific and prognosis-related ones. One gene may have up to five types of AS events (red dotted line) that are differentially expressed and survival-associated in ccRCC. (b–h) Forest plot which displays the top 20 (if available) most significantly survival-associated AS events in each type, respectively. *P*-values of univariate Cox regression analyses are indicated by the color scale by the side. Unadjusted hazard ratios (boxes) and 95% confidence intervals (horizontal lines) are depicted in the plot

Moreover, we attempted to assess the prognostic ability of these identified aberrant AS events. By using univariate Cox regression analyses, we identified a total of 2100 survival-associated AS events from 1666 parent genes (*p*-value < 0.05), including 1975 unfavorable prognostic splicing events (hazard ratio (HR) > 1, *p*-value < 0.05) and 125 favorable prognostic ones (HR < 1, *p*-value < 0.05). To further quantify the interactions among these aberrant survival-associated events, we applied Upset plot and revealed that some genes clearly had up to five types of AS events which were both cancer-specific and prognosis-related (Figure 3a). For example, AD, AP, ES, RI and AA events of DMKN (red dotted line) were all significantly associated with patient OS in ccRCC. NDRG2 and POFUT2 (green dotted lines) could possess four types of prognostic AS events. The exact number of aberrant splicing events indicating poor prognosis far exceeded the number of those indicating good prognosis, which were consistent with the results from differential spliced analysis and appeared to confirm a major oncogenic role of aberrant AS in initiation and progression of ccRCC. Moreover, for each AS type, the top significant biomarkers were selected and visualized in forest plots (Figure 3(b–h)). Interestingly, most of them were unfavorable prognostic factors, suggesting potential positive correlations between aberrant splicing variants and carcinogenesis.

**Figure 4.**
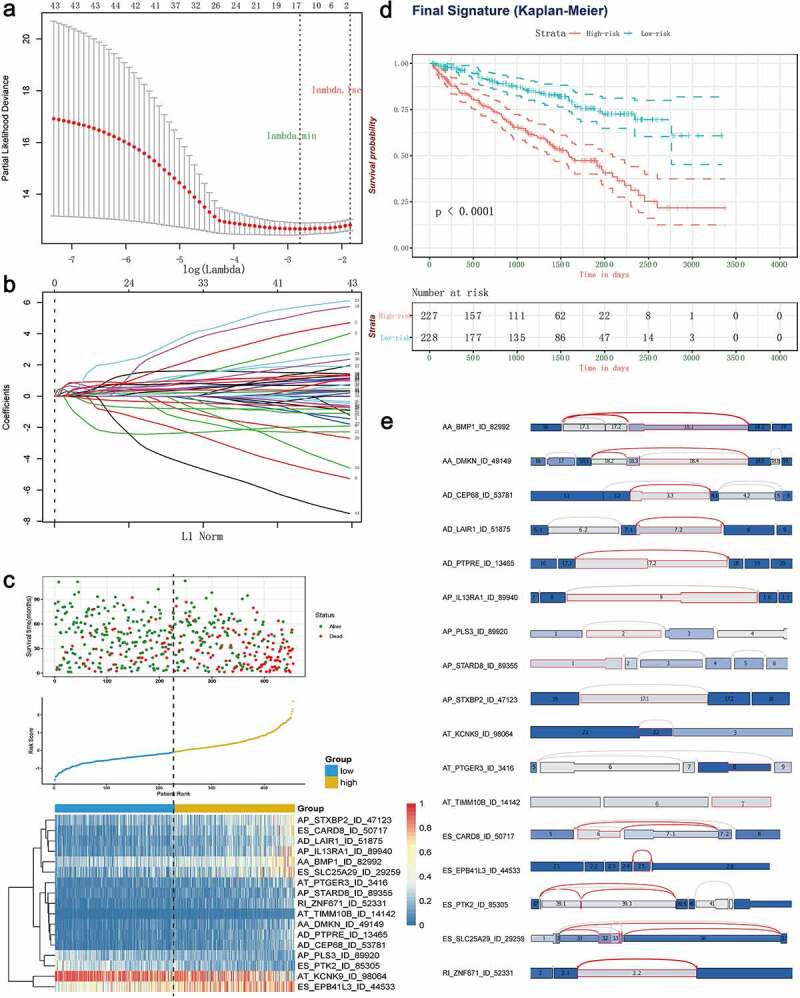
Least absolute shrinkage and selection operator (LASSO) penalized Cox model to build final prognostic model by combining all types of AS events within each signature. (a) The selection of tuning parameter (lambda) in the LASSO-penalized Cox regression model via 10-fold cross validation for constructing final prognostic signature. (b) The shrinkage procedure of LASSO regression coefficients to determine the optimal number of features in final model. (c) The distribution of patient survival status ranked by corresponding risk score, the splicing pattern of specific AS events included in final signature. (d) The Kaplan-Meier survival curves of overall survival between high-risk and low-risk patients, which are divided by the median value of final risk score. (e) The splicing graph of 17 specific AS events included in final AS signature, with corresponding splicing patterns illustrated by red lines. The thin exon region represents the protein-coding region, and the thick region represents the untranslated region

**Figure 5.**
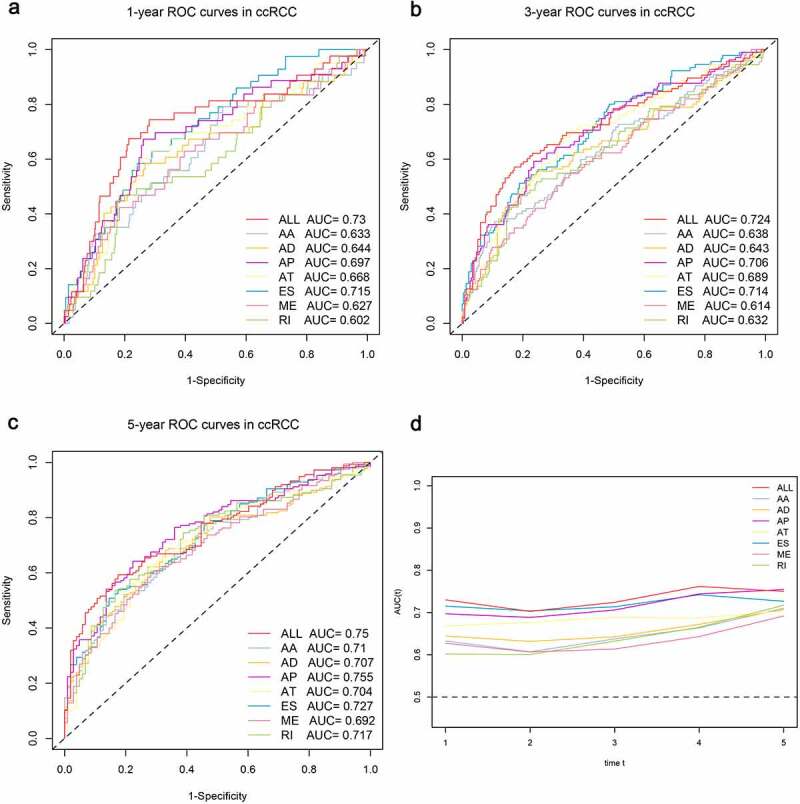
Time-dependent ROC curves for comparable analyses on predictive performance of the models in ccRCC cohort, including the seven AS signatures and final prognostic signature. (a–c) Time-dependent ROC curves of each AS signature and final signature for predicting overall survival of ccRCC patients in 1, 3 and 5 years. (d) The comparable area under curve (AUC) values spanning 1 year to 5 years according to each signature

Next, the top 20 most significantly survival-associated events in each AS type (only 5 ME events available) were chosen as candidates for establishing prognostic signature (Figure 3(b–h)). To screen out AS events with the greatest prognostic value, we applied LASSO-penalized regression analysis with 10-fold cross validation in each AS type, separately. LASSO-penalized model is a machine learning method, selecting the most important features by weighing the number of variables and model fitness iteratively. For instance, the coefficients of majority of AA events have been shrunk to zero in procedure of model fitting (Supplementary Fig. S3A), with only 4 AA events left with nonzero status based on ‘lambda.min’ criteria (Supplementary Fig. S2A). As results, we have generated seven prognostic signatures, comprised of 4 AA events, 6 AD events, 10 AP events, 7 AT events, 11 ES events, 4 RI events and 3 ME events, respectively (Supplementary Fig. S2 and S3). As demonstrated (Supplementary Fig. S4), patients were stratified into low-risk and high-risk subgroups based on the corresponding risk score, utilizing the median cutoff calculated in the entire cohort. The distribution of patients’ survival time and status, heatmaps of selected splicing events were also displayed. The Kaplan-Meier survival curves also indicated that patients of high-risk were with significantly poor OS compared than those of low-risk (Supplementary Fig. S5, *p*-value < 0.0001). Eventually, with the combination of all available types of AS events that formed signatures, we screened 17 particular AS events via LASSO-penalized Cox method (Figure 4a and 4b) and further constructed a final AS-based panel by fitting multivariate Cox regression model (Figure 4c). In addition, the median risk score also served as the cutoff point for assigning ccRCC patients into low- and high-risk groups. As shown in Figure 4d, the high-risk patients showed a 3.13-fold higher risk (95% confidence intervals (CI): 2.18–4.50, *p*-value < 0.0001) than low-risk patients. Therein, the detailed splicing pattern of each splicing event was depicted in Figure 4e. Encouragingly, time-dependent ROC curves (Figure 5(a–c)) also revealed that the AUC values of established AS panel were 0.73 at 1 year, 0.724 at 3 years and 0.75 at 5 years, confirming the predictive potential of our AS panel for both short-term and long-term prognosis with AUCs being robust above 0.7 across time (Figure 5d). The detailed formula of each AS signature is listed in Supplementary Table S1.

## Functional enrichment results of identified aberrant survival-associated AS events

It was evident that AS has a profound effect in altering transcript architecture and further modifying corresponding protein via deleting or adding functional domains. Thus, analyzing the influencing of aberrant AS on their encoding proteins might give us a better understanding and direction of the roles of aberrant events in the splicing machinery of ccRCC. In our research, 1666 genes from 2100 both ccRCC-specific and OS-related splicing events were sent for functional enrichment analysis, including GO and KEGG pathway. A total of 48 GO-CC categories, 60 GO-BP and 25 GO-MF terms were identified as significantly enriched ones (FDR < 0.05), most of which were closely related to mitochondrial and energy metabolic activities, such as mitochondrial matrix (FDR < 0.0001), generation of precursor metabolites and energy (FDR = 0.003), and mitochondrial inner membrane (FDR = 0.002). Additionally, specific biological processes closely associated with ccRCC, such as focal adhesion (FDR = 0.002), RNA splicing (FDR = 0.015), and GTPase regulatory activities, were significantly enriched in these parent genes occurred aberrant AS events (Supplementary Fig. S6). Besides, several KEGG pathways that were experimentally validated participating in ccRCC progression or metastasis, including central carbon metabolism in cancer (FDR = 0.011), ubiquitin mediated proteolysis (FDR = 0.011), base excision repair (FDR = 0.043) and apoptosis (FDR = 0.016), were also revealed. Of note, both KEGG and GO enrichment analyses suggested the regulation of autophagy could be a potential mechanism of splicing regulatory in ccRCC. Taken together, above functional enrichment results not only provided novel clues for further exploring the underlying mechanisms upon aberrant prognosis-related events, but also confirmed the reliability of our screening procedure in turn.

## Altered biological pathways between the high- and low-risk ccRCC patients

The strong risk stratification ability of our 17 AS-based panel for ccRCC patients could be attributed to the oncogenic pathways and biological process that participate in ccRCC regulation during the tumor development or metastasis. Hence, we performed GSEA to elucidate potential biological functions of the final AS panel (Supplementary Fig. S7A and Fig. S7B). As results, we observed that genes highly expressed in the high-risk group showed significantly enriched in ‘P53 signaling transduction’ and ‘MAPK signaling pathway’, which have been widely acknowledged being associated with the progression of ccRCC. Furthermore, several cancer-related pathways, such as the cell cycle regulation, response to DNA damage and neutrophil chemotaxis, were also up-regulated in high-risk patients, while the high-risk correlated genes were revealed to be associated with down-regulation of autophagy, which was in accordance with the KEGG enrichment results above. In summary, our GSEA analysis implied that the final 17 AS-based signature might be involved in crucial ccRCC-related pathways and their functional dysregulation subtly influences the OS of ccRCC patients.

## The independent predictive power of final AS panel for ccRCC patients

**Figure 6.**
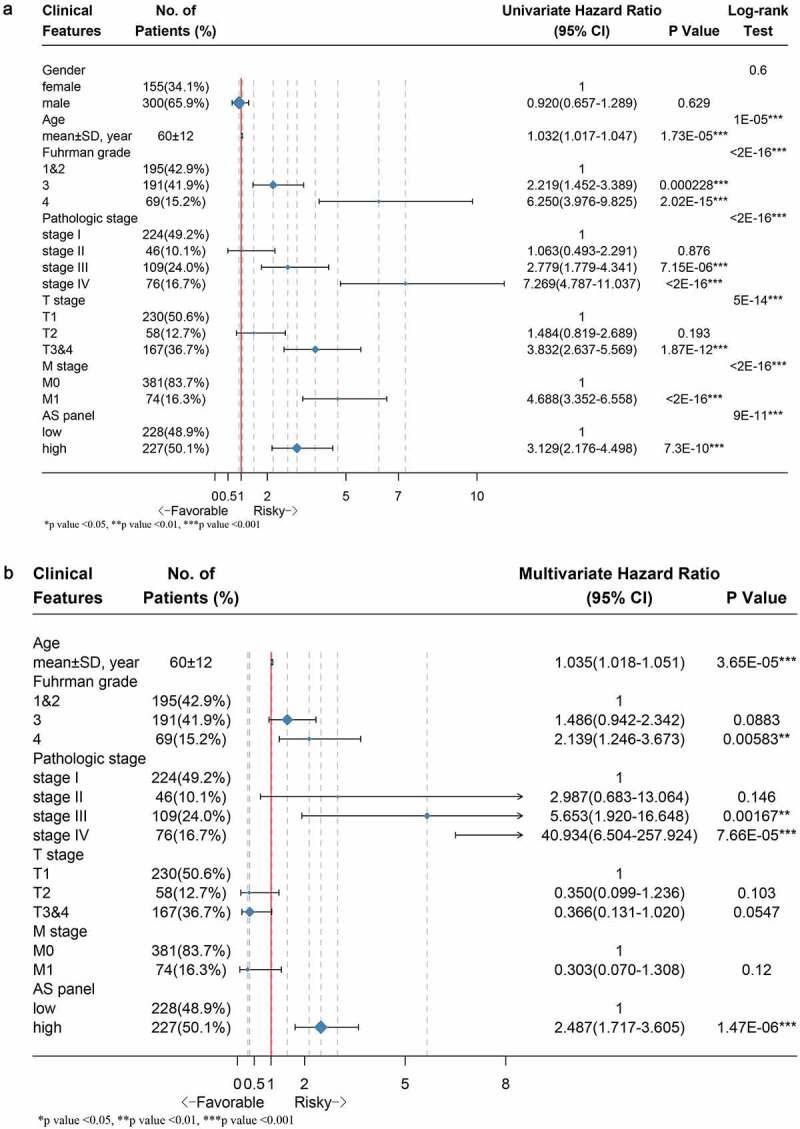
Forest plots displaying the results from univariate and multivariate Cox regression analysis to validate independent prognostic value of the final model. (a) Univariate Cox analysis of available clinicopathologic features and final AS signature on overall survival. (b) Multivariate regression analysis of the relation between final AS prognostic model and clinicopathologic characteristics regarding prognostic value. The length of horizontal line corresponds to the 95% confidence interval (CI), and the relative size of box is in proportional to the sample size. The red vertical line indicates HR of 1.0. (*p-value < 0.05, **p-value < 0.01 and ***p-value < 0.001)

We performed univariate followed by multivariate Cox hazard regression analyses of data in TCGA ccRCC cohort in order to further investigate whether the final 17 AS-based panel was an independent prognostic factor, where the AS panel was treated as a binary variable. The univariate analysis results suggested that age, Fuhrman grade, pathological stage, T stage, M stage and final AS panel were all remarkably correlated with the OS of ccRCC patients (Figure 6a). Therefore, those significant factor were included in a multivariate analysis, which showed that the AS panel (HR = 2.487; 95% CI: 1.717–3.605; *p*-value = 1.47e-06), age, Fuhrman grade, pathological stage were four independent prognostic factors when adjusted by those risk factors (Figure 6b).

Moreover, in order to investigate the prognostic value of our AS panel in stratified cohort, we classified patients into various subgroups based on age, gender, pathological stage, Fuhrman grade, T stage and M stage then we performed stratification analysis. As shown (Supplementary Fig. S8), the AS panel identified patients with distinct prognoses in all cohorts analyzed, thus confirming its robustness for independently predicting ccRCC prognosis.

## Development and apparent performance of a nomogram based on the final AS panel

**Figure 7.**
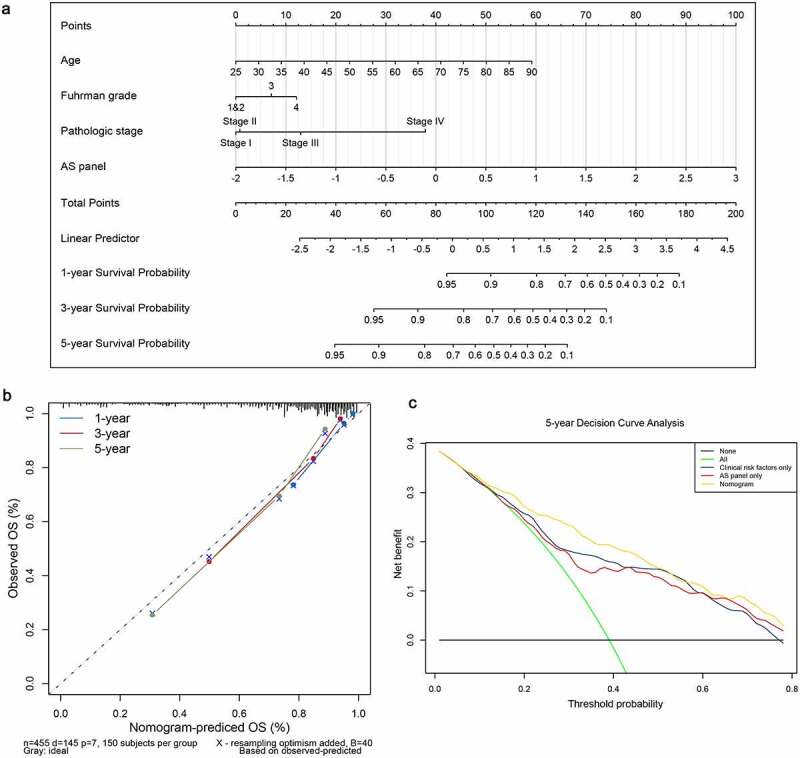
Construction and evaluation of a clinical predictive model at aspects of calibration and clinical usefulness. (a) A nomogram for predicting the survival probability of 1-, 3- and 5-year overall survival for ccRCC patients, which was built based on four independent prognostic factors in ccRCC. (b) Plot depicts the calibration of the nomogram based on overall survival in terms of agreement between predicted and observed 1-, 3- and 5-year clinical outcomes. The dashed 45-degree line indicates the ideal prediction. (c) Decision curve analysis for evaluating 5-year clinical usefulness of nomogram in ccRCC patients

A second multivariate Cox regression model that incorporated the above independent predictors, including the final AS panel, age, Fuhrman grade and pathological stage, was developed and presented as the nomogram (Figure 7a). It substantiated that the AS panel comprised of 17 AS events contributes the most risk points (ranging from 0 to 100), whereas the other clinicopathological characteristics contribute much less. The C-index for OS prediction was 0.816 with 1000 bootstrap replicates (95% CI: 0.784–0.848), suggesting derived nomogram showed good predictive discrimination ability. And the calibration plot showed that the bias-corrected lines were close to the ideal line (45° line), indicating an optimal agreement between the survival prediction by our nomogram and actual observation (Figure 7b). Moreover, we conducted DCA analysis to determine the real-world clinical usefulness of the AS-clinicopathological nomogram by quantifying the net benefits against a range of threshold probabilities in ccRCC cohort. And the DCA results are presented in Figure 7c, which showed that using our AS-based nomogram would add more benefits in prognosis prediction than either treat-all-patients or treat-none scheme if the threshold probability of a patients or clinician is more than 10%. Within this range, the net benefits were comparable, with several overlaps, based on the final AS panel and the model with clinicopathological factors integrated only, further convincing the AS panel is non-inferior to clinical features. Besides, the clinical usefulness of the nomogram significantly overwhelmed the clinical risk factors. In a word, these findings suggested that the derived nomogram was a better prognostic model for predicting short-term and long-term survival probability in ccRCC patients than individual clinical indicators.

## The prognostic SFs and construction of splicing regulatory network

**Figure 8.**
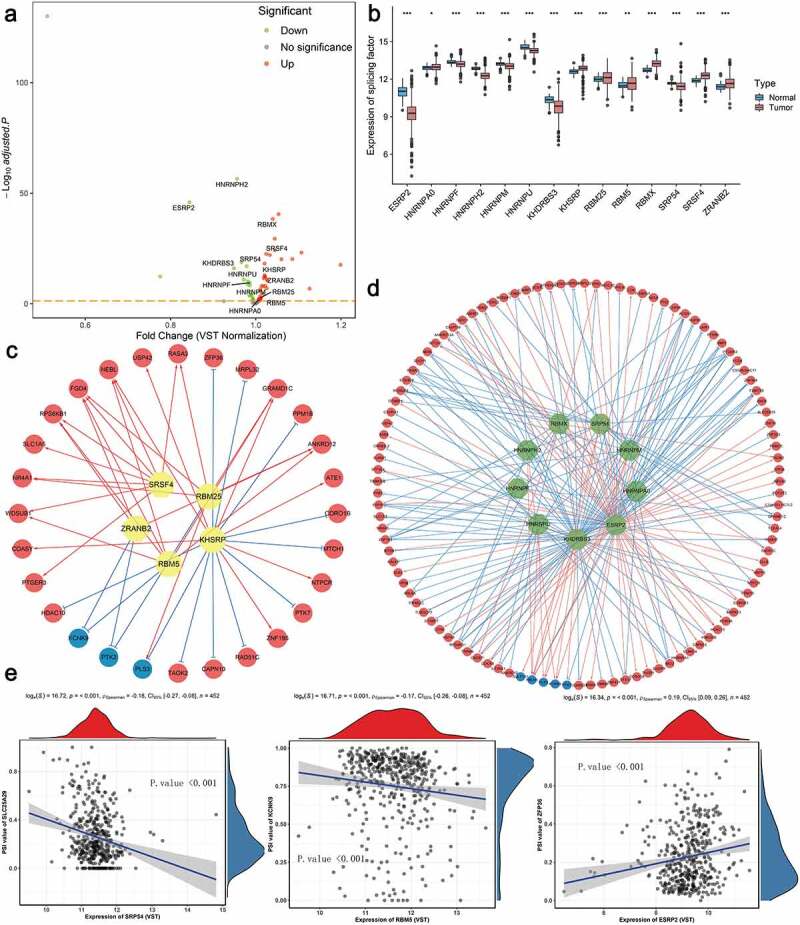
Identification of aberrant prognostic splicing factors (SFs) and construction of SF-AS splicing regulatory network in ccRCC. (a) Volcano plot showing the differentially expressed SFs between tumor and normal adjacent cancer tissues. The red points indicate the upregulated SFs in tumor samples, whereas the green points represent the downregulated SFs in tumor tissues. The gene symbols of survival-associated ones are further labeled in the plot. (b) The difference of relative expression levels of aberrant prognostic SFs between ccRCC samples and normal samples. Boxes encompass 1st–3rd interquartile range of expression values, black lines in boxes represent the median values, whiskers indicate 1.5 times the interquartile range, and the black points indicate the outliers. (*p-value < 0.05, **p-value < 0.01 and ***p-value < 0.001). (c–d) Splicing regulatory networks, showing the positive (red line) or negative (green line) correlation between these SFs (yellow node indicates the risky one, while green node indicates the protective one) and the most significant AS events (red node represents the adverse prognosis event, while blue node represents favorable prognosis event). (e) Representative correlation pairs in SF-AS regulatory network

It has been proved that the global dysregulation of AS events could be orchestrated by a limited number of SFs, especially in ccRCC. The normalized level 3 RNA-seq profiles of TCGA ccRCC samples identified 53 SFs whose expression levels differed significantly between tumor tissues and adjacent normal tissues (Figure 8a). According to the thresholds, 14 SFs meeting the cancer-specific and prognosis-related properties were further screened as candidates (Figure 8b and Supplementary Fig. S9). Next, to systematically decipher the cancer-specific splicing regulatory connections in ccRCC, splicing regulatory networks, which enrolled the significant relationships (adjusted *p*-value < 0.05) among these 14 SFs, including 5 risky ones (yellow polygons) and 9 SFs indicating favorable prognosis (green polygons), and 110 most significantly prognostic AS events comprised of 105 risky ones (red dots) and 5 favorable ones (blue dots), were finally built, respectively. As shown in Figure 8(c–d), the majority of genes could be synergistically or competitively regulated by different SFs via binding the same transcript regions, further revealing the intratumoral complexity and intricate regulating modification in driving initiation, progression and metastasis of ccRCC. And these kinds of phenomena also account for the reasons that transcript could yield several different protein isoforms which functioned opposite biological behaviors, especially in ccRCC. More interestingly, we found that most poor prognosis-related splicing events (red dots) were positively correlated (red lines) with risky SFs (yellow polygons) but negatively regulated (blue lines) by favorable SFs (green polygons). Representative scatter plots presenting the significant correlations between expression levels of particular SFs and PSI scores of specific AS events are displayed in Figure 8e. For instance, the expression of ESRP2 was positively correlated with AD event of ZFP36, while the ES of SLC25A29 could be negatively regulated by SRP54.

## The ccRCC subtypes associated with distinct clinical outcomes

**Figure 9.**
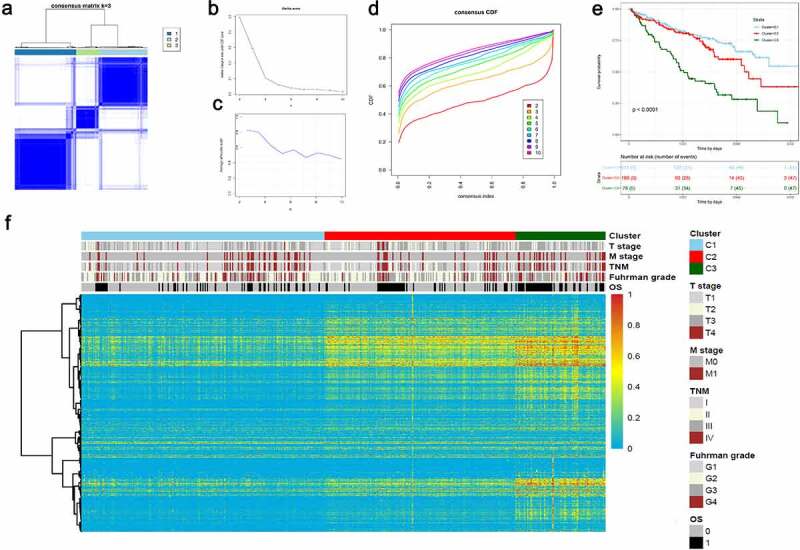
Unsupervised consensus cluster analysis revealed distinct ccRCC classifications associated with different outcomes and clinicopathological characteristics. (a) Consensus matrix heatmap defined three AS clusters based on aberrant prognostic splicing events. (b–c) The Elbow plot and Silhouette statistics to determine the optimal number of consensus clusters. (d) The distribution of CDF curves of the consensus scores (*k* = 2–10) in ccRCC cohort. (e) The Kaplan-Meier survival analysis of ccRCC patients within different clusters. *P*-value was calculated from log-rank test. (f) Heatmaps depicting the relationships among molecular subtypes, splicing patterns and clinicopathological characteristics

According to the Elbow plot (Figure 9b) and Silhouette statistics (Figure 9c), the consensus cluster approach helped us to identify three distinct molecular subtypes (*k* = 3) based on the splicing patterns of these AS candidates (Figure 9d). Consequently, three clusters of ccRCC patients were determined as follow: C1 (*n* =  211, 46.4%), C2 (*n* =  166, 36.5%) and C3 (*n* =  78, 17.1%) (Figure 9a). The survival analyses suggested that AS clusters were significantly associated with distinct prognostic patterns (*p*-value < 0.0001, Figure 9e), among which C3 subtype was associated with poor outcome. Additionally, the heatmap also depicted the associations of clusters with clinicopathological features, which revealed that tumors classified as C3 were more frequently holding higher histological grades and metastasis properties (Figure 9f).

## Disscussion

As changeable and possibly heritable post-transcriptional process, AS mechanisms offer promising clues for the therapeutic strategies in various disease, including many cancers [11]. To data, accumulated evidence indicates that the AS process could make crucial contribution to the initiation, progression and metastasis of cancer, especially in renal cell carcinoma. For instance, DCLK1-ASVs, as an alternate splice variant of DCLK1, plays a stem cell supportive role in kidney cancer via driving self-renewal and drug resistance to chemotherapy [38]. VEGF is widely regarded the most potent growth factor for tumor neovasculature, while in-depth study has revealed that VEGF_165_b, a differential splicing isoform from exon 7 to 3ʹ untranslated region, is down-regulated in renal cell carcinoma and contributes to anti-angiogenesis [39]. Similarly, PKM2, rather than PKM1, has been reported to promote different aspects of ccRCC progression, including cell proliferation, invasion and migration in vitro [40]. Meanwhile, the involvement of SFs is also reflected in the investigation of aberrant AS regulation in ccRCC. The pro-tumorigenic function of SF3B3 can be partly mediated by up-regulation of EZH2Δ14, since the EZH2Δ14 acts as a suppressor for the expression of EZH2, which is commonly recognized as a tumor-suppressive gene [41]. Accordingly, AS events have been considered as multifaceted carcinogenesis hallmark of ccRCC, convincing that splicing events could be preeminent biomarkers for further investigation. Even so, painstaking exploration of therapies concerning aberrant AS, including clinical trials, are nevertheless under way [14,42]. To our best knowledge, our present study is a relatively comprehensive attempt to decipher the untapped mechanisms of AS in ccRCC so far.

The prevalence of ccRCC, accounting for approximately 75% of primary kidney cancer, is increasing annually with recent implementation of screening program. Despite the advance of surgical procedures, such as partial or total nephrectomy, more than 20% postoperative patients suffering from ccRCC will develop distant metastasis [3,43]. What’s worse, adjuvant systematic therapies, including chemotherapy, radiotherapy and immunotherapy, have failed to show an obvious survival benefit for postoperative patients, highlighting the drug resistance and financial burden as problems worth pondering [1,44]. In this aspect, novel molecular biomarkers that could reliably evaluate disease progression and patient prognosis would have tremendous potential in guiding therapeutic strategies and clinical management of ccRCC patients. Taking the potential influence of aberrant splicing events in renal carcinogenesis into account, it is reasonable to consider the AS events in to increase the effectiveness of adjuvant therapy. In this study, using the well-established public, large-scale multi-omics sequencing cohort, we proposed a robust, individualized AS panel that can estimate OS probability in ccRCC patients based on 17 aberrant splicing events. Attributed to the combination of all the seven types of AS events, the AUC values of ROC curves for this model can keep above 0.7 spanning 1 year to 5 years, suggesting enhanced power and great potential in short-term and long-term prognosis prediction for ccRCC patients. Moreover, our prognostic AS panel could stratify clinically defined groups of patients (e.g. age, TNM stage and Fuhrman grade) into subgroups with distinct clinical outcomes.

Actually, to illustrate the clinical value of AS events in cancer, several investigators have performed genome-wide analyses of splicing events in non-small cell lung cancer, gastrointestinal adenocarcinoma and ovarian cancer [45–47]. With the rapid development of high-throughput sequencing technologies over the last decades, preliminary success has also been gained in insight into the involvement of splicing patterns in ccRCC. Recently, by integrating whole-exome sequencing, RNA-seq and copy number variation analyses, Lehmann et al analyzed 282 ccRCC patients and revealed that 915 probable splicing quantitative trait loci with tumor-specific splicing pattern were identified, and that are involved in processes relevant for tumor growth and cancer progression [48]. However, few studies have broken the gap between aberrant AS events and clinical relevance in a systematic way. And it still remains to be unsolved problem that what extent alteration of AS pattern could change the clinical outcome among ccRCC patients. Despite a series of strict criteria were conducted in the process of sample screening and biomarker filtration, more AS events, consisting of a total of 2100 aberrant splicing events occurred in 1666 parent genes, were finally identified in present study, meeting the cancer-specific and prognosis-related properties. Moreover, our exploration of AS patterns broadens the understanding of traditionally conceptual prognostic biomarker, which serves as a powerful complementary to verify the link between AS and ccRCC.

Furthermore, functional enrichment analyses, including GO, KEGG and GSEA analysis, provided more opportunities for deciphering the largely untapped mechanisms which our identified AS events might participate in. Intriguingly, there were several intersections among our enrichment results and preliminary investigation. The majority of enriched biological behaviors were metabolism-related and the autophagy-related pathways were revealed as the significant overlap among GO, KEGG and GSEA results. Autophagy, as an intracellular self-degradative process for capturing and recycling degraded components with the assistance of autophagosome and lysosome, plays crucial roles in maintaining metabolism and homeostasis [49]. More importantly, the modulation of autophagy involves in dual-sided roles within cancer microenvironment, which could facilitate tumorigenesis via promoting proliferation and mediate tumor suppression via inducing cell death and apoptosis [50]. ccRCC is distinguished by inactivating mutations in VHL (von Hippel-Lindau tumor suppressor), leading to constitutive activation of the hypoxia-inducible factors (HIFs) and induction of a hypoxia response transcription signature. Hall et al. found TRPM3 and miR-204 established a regulatory circuit which could promote the growth of ccRCC and stimulate MAP1LC3A (LC3A) and MAP1LC3B (LC3B) autophagy. VHL represses TRPM3 directly through miR-204 and indirectly through another miR-204 target [51].

Moreover, correlation analyses were performed in a preliminary exploration to elucidate the underlying splicing mechanisms in ccRCC. Differed SFs are in competition for the same splicing sites of parent genes, which at least partly, deciphered that gene transcripts could yield distinct splicing isoforms driving opposite biological behaviors. More intriguingly, almost all the risky SFs exerted significant positive regulatory effects on the poor prognostic AS events, whereas most favorable splicing events were negatively correlated with unfavorable splicing events. In overall, our SF-AS regulatory network provided new approaches to address splicing regulatory patterns involved in ccRCC. Therein, several SFs, revealed with significant aberrant expression and prognostic values in our cohort, represented consistent tendency with previous reports. ESRP2 is a kind of splicing regulator specifically expressed in epithelial cells, regulating of the function of cytoskeleton and cell motility. Mizutani et al. reported that the expression of some ESRP-target exons was related with good prognosis in ccRCC and with the expression of RNF111, a molecular which interacted with ESRP2, induced polyubiquitination and suppressed ccRCC tumor growth in a coordinated manner [52]. hnRNPs regulate almost all levels of expression of apoptotic genes and play a vital role in DNA repair, telomere biogenesis, cell signaling, mRNA stability, alternative splicing, and protein degradation. Through these key cellular functions, individual hnRNPs have a variety of potential roles in tumor development and progression including the inhibition of apoptosis, angiogenesis and cell invasion [53].

Tumor heterogeneity highlighted the limitations of traditional risk factors. They may be insufficient for proper tumor characterization and classification, leading to difficulties for the prediction of disease progression, therapeutic response and prognosis. Nowadays, powerful tools, including high-throughput and genome-wide profiling methodologies, have led to significant progress in understanding of ccRCC [54]. Here, we demonstrated that our AS panel could serve as an independent prognostic factor for OS prediction in ccRCC patients. Besides, we further leveraged the complementary value of AS panel and other conventional clinicopathological features (age, Fuhrman grade and pathologic stage). Consequently, our nomogram could be a valuable prognostic scoring system for clinical decision-making of practitioners, represented as a robust and reliable tool for prognosis prediction in ccRCC.

However, several limitations of our study were still required to be clarified. Firstly, a major shortcoming of our work is due to its retrospective nature, and clearly, multicentre prospective studies with large sample size are warranted to further validate our findings, especially these clinical relevant splicing events. Secondly, owing to the existence of missing value of clinicopathological characteristics and a fraction of splicing events in our inclusive cohort, the statistical power in integrated prognostic model could be inevitably decreased. Thirdly, due to the emerging roles and relatively complicated mechanisms of aberrant splicing in tumorigenesis, only a few irregular spliced isoforms within the enormous splicing events detected have been verified as direct contributors to tumors, with further functional experiments needed in ccRCC. In addition, many uncertainties still exist concerning detailed biological processes of SFs in recognizing, binding and regulating mRNA splicing, for only preliminarily in silico exploration on their surface correlations in the current work.

## Conclusion

In summary, for the first time, through comprehensively analyzing the genome-wide splicing events, we identified an AS panel composed of 17 aberrant AS events in ccRCC. The interpretation of aberrant AS patterns and corresponding regulatory networks may motivate a novel insight in the oncogenic process of ccRCC and provide potential therapeutic targets that require further validation.

## Supplementary Material

Supplemental Material

## Highlights

- Genome-wide ccRCC-specific AS patterns were profiled in a large-scale population.
- Seventeen AS events were chosen to build a prognostic model with high performance in ccRCC.
- Prognosis-related SF-AS regulatory network deciphered splicing mechanisms in ccRCC.
- We revealed three ccRCC clusters with distinct splicing and clinicopathological patterns.
