# Supplementary material for: Novel insights into clear cell renal cell carcinoma prognosis by comprehensive characterization of aberrant alternative splicing signature: a study based on large-scale sequencing data
Source: Bioengineered. 2021 Mar 30;12(1):1091–110. doi: 10.1080/21655979.2021.1906096 (PMC8806224; doi:10.1080/21655979.2021.1906096)
Supplement: Supplemental Material [file KBIE_A_1906096_SM0788.zip › Document.rtf]

Supplementary Figure S1. The Upset plot depicting the parent gene intersection among the seven types of AS events which were identified in ccRCC tissues.

Supplementary Figure S2. LASSO-penalized Cox regression analysis with 10-fold cross validation to reduce dimension and select key features using the top significant aberrant survival-associated splicing variants in each AS type. The selection of tuning parameter (lambda) in the LASSO-penalized Cox regression model for constructing each AS-based prognostic signature, including (A) AA, (B) AD, (C) AP, (D) AT, (E) ES, (F) RI and (G) ME type. The partial likelihood deviances (y-axis) from the cross-validation procedure of LASSO regression are plotted as a function of log(lambda) (lower x-axis). Dynamically changing number along the upper x-axis indicates the number of variables kept in final model with given lambda. Dashed vertical lines from left to right define the log of the optimal value of lambda via "minimum" and "1-fold standard error" criteria, respectively. In our study, we applied "minimum" criteria for feature selection, which minimizes the prediction error and will give the most accurate model.

Supplementary Figure S3. LASSO-penalized Cox regression analysis to shrink the coefficients of key features using the top significant aberrant survival-associated splicing variants in each AS type. The shrinkage procedure of LASSO regression coefficients to determine the optimal number of features in each AS signature, including (A) AA, (B) AD, (C) AP, (D) AT, (E) ES, (F) RI and (G) ME. In these procedures, only the most significant indicators are kept in the final model, whereas the coefficients of some less contributive features are shrunk to zero. The dashed vertical lines are generated by the regression analyses (in Supplementary Figure S2) based on the minimum criteria with 10-fold cross validation, separately.  

Supplementary Figure S4. The distribution of patient survival status ranked by corresponding risk score, the splicing pattern of particular AS events included in each signature, including (A) AA, (B) AD, (C) AP, (D) AT, (E) ES, (F) RI and (G) ME. Upper two-thirds panel: risk score distribution with patient survival status. The x-axis is ranked by the values of corresponding risk score. Yellow color indicates the patients at high-risk while blue color indicates low risk. And red dot represents patient is dead while green dot represents survive. Lower third panel: Heatmap showing the PSI range of corresponding AS events.   

Supplementary Figure S5. Kaplan-Meier survival analyses of overall survival between high-risk and low-risk patients, which are divided according to median risk score of the (A) AA, (B) AD, (C) AP, (D) AT, (E) ES, (F) RI and (G) ME signatures in ccRCC. The x-axis indicates the overall survival time in days, and the y-axis indicates the survival probability. The red curves represent the high-risk subgroup while the green curves represent the low-risk group.

Supplementary Figure S6. Functional enrichment analyses of parent genes which occurred aberrant survival-associated AS events in ccRCC patients, revealing the potential orchestration of AS in tumorigenesis. (A-C) Bubble diagrams showing the top significant terms from Gene Ontology (GO) analyses of corresponding genes occurred aberrant survival-associated AS events, at aspect of (A) biological process, (B) cellular component and (C) molecular function. (D) The most significant pathways identified with Kyoto Encyclopedia of Genes and Genomes (KEGG) pathway analysis.


Supplementary Figure S7. Gene set enrichment analysis (GSEA) reveals the underlying mechanisms of splicing events included in final prognostic signature. (A) Significant GSEA results of hallmark gene sets named "c2.cp.v6.2.symbols" downloaded from Molecular Signatures Databases. (B) GSEA analysis delineates the biological processes related to final signature using the "c5.bp.v6.2.symbols" gene sets downloaded from Molecular Signatures Databases.   

Supplementary Figure S8. Stratification Cox analyses of final AS signature in ccRCC cohort, validating its independent roles in predicting prognosis. Survival curves of overall survival for ccRCC patients in subgroup stratified by (A) age, (B) gender, (C) pathologic stage, (D) Fuhrman grade, (E) T stage, (F) M stage, showing that the survival of patients was significantly poorer in patients with high risk in stratified cohorts.

Supplementary Figure S9. Kaplan-Meier survival analyses of aberrant prognostic splicing factors (SFs) in ccRCC with cutoff point identified. The optimal cutoff value of each particular SF is displayed above the survival curve, respectively.

Supplementary Table S1. The detailed formula of each AS-based prognostic signature in ccRCC, showing how to calculate the individualized risk score for each patients, respectively.
