# Supplementary figures and images for: Novel insights into clear cell renal cell carcinoma prognosis by comprehensive characterization of aberrant alternative splicing signature: a study based on large-scale sequencing data
Source: Bioengineered. 2021 Mar 30;12(1):1091–110. doi: 10.1080/21655979.2021.1906096 (PMC8806224; doi:10.1080/21655979.2021.1906096)

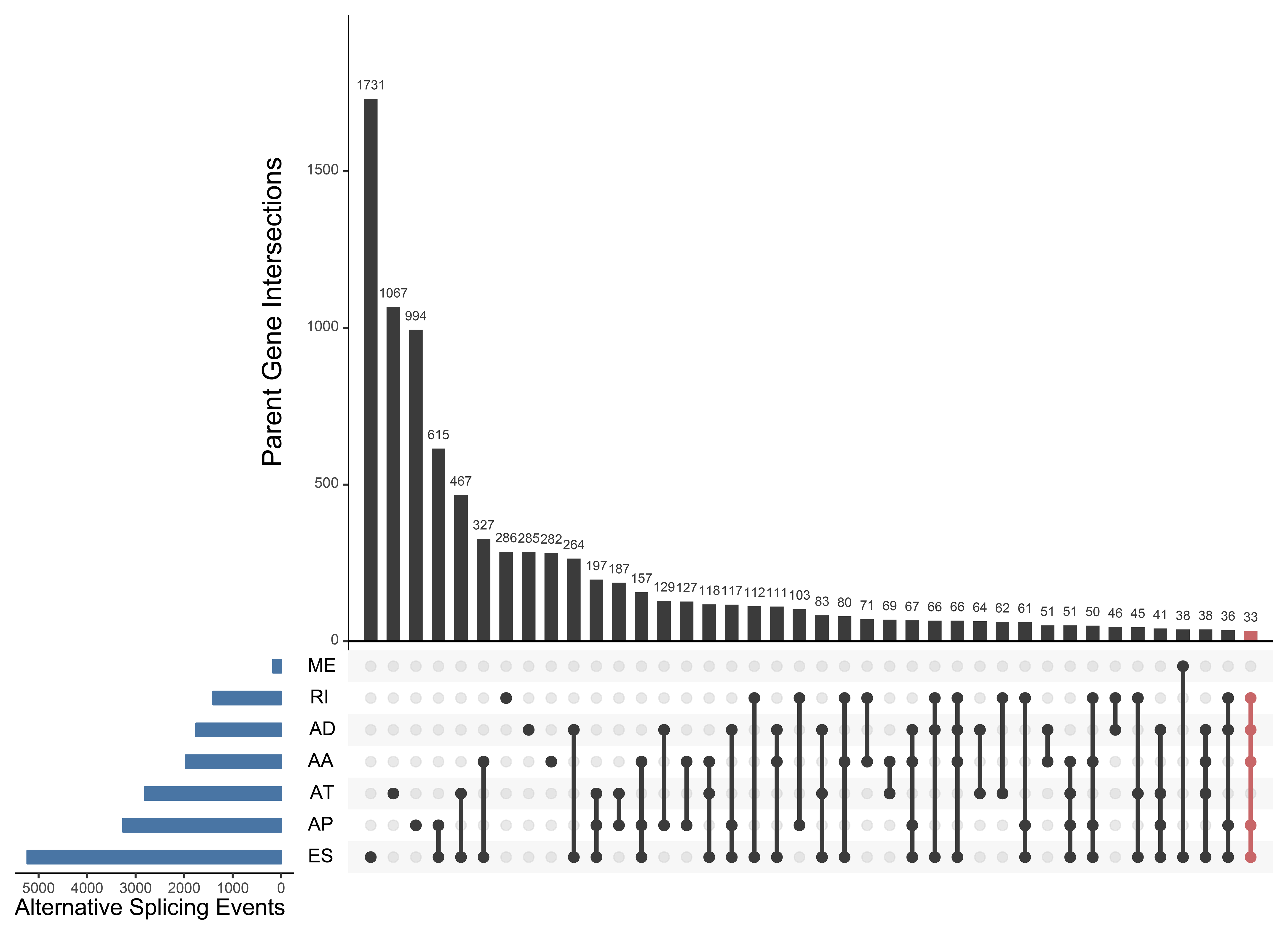

Supplement: Supplemental Material [file KBIE_A_1906096_SM0788.zip › Supplementary Figure S1.tif]

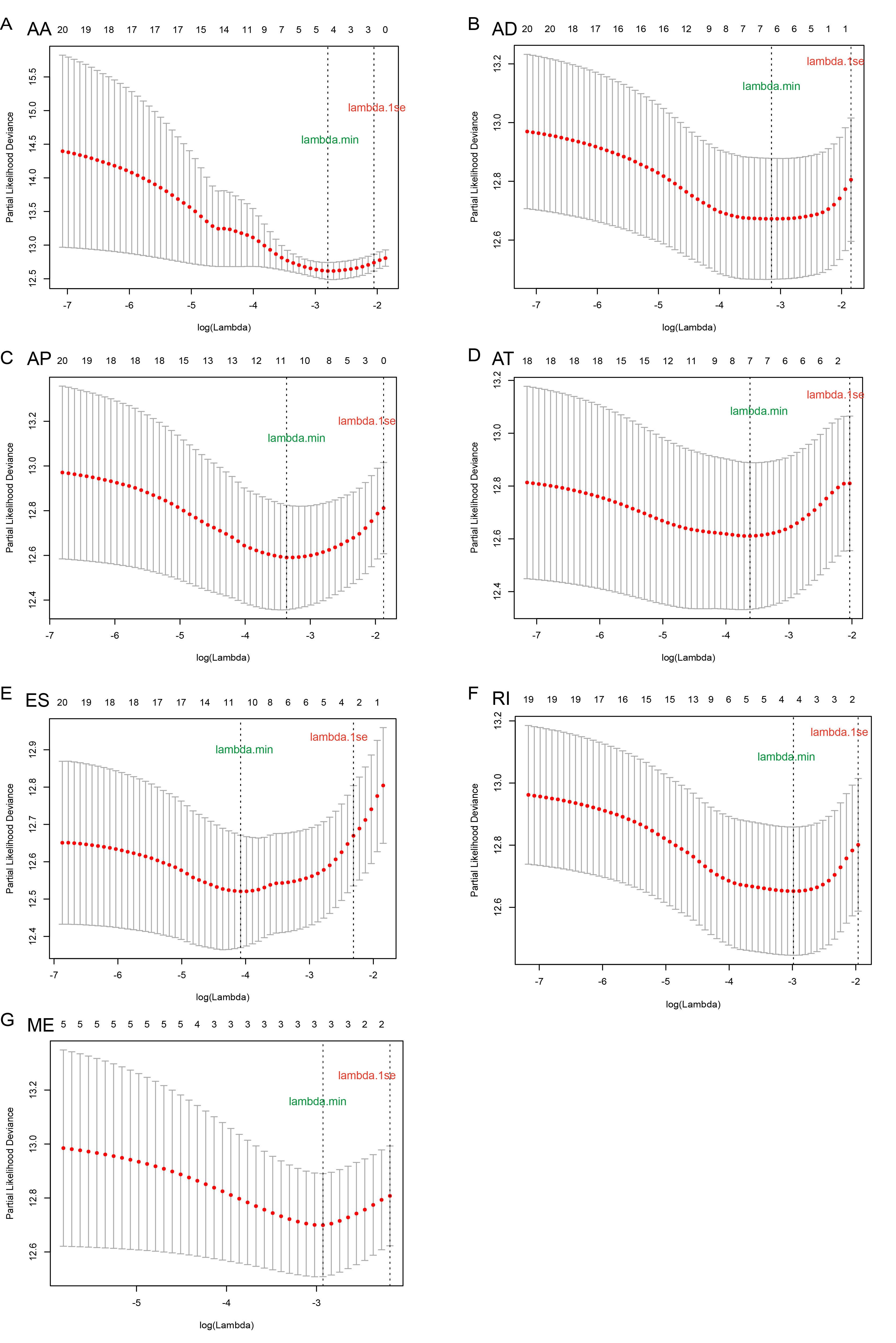

Supplement: Supplemental Material [file KBIE_A_1906096_SM0788.zip › Supplementary Figure S2.tif]

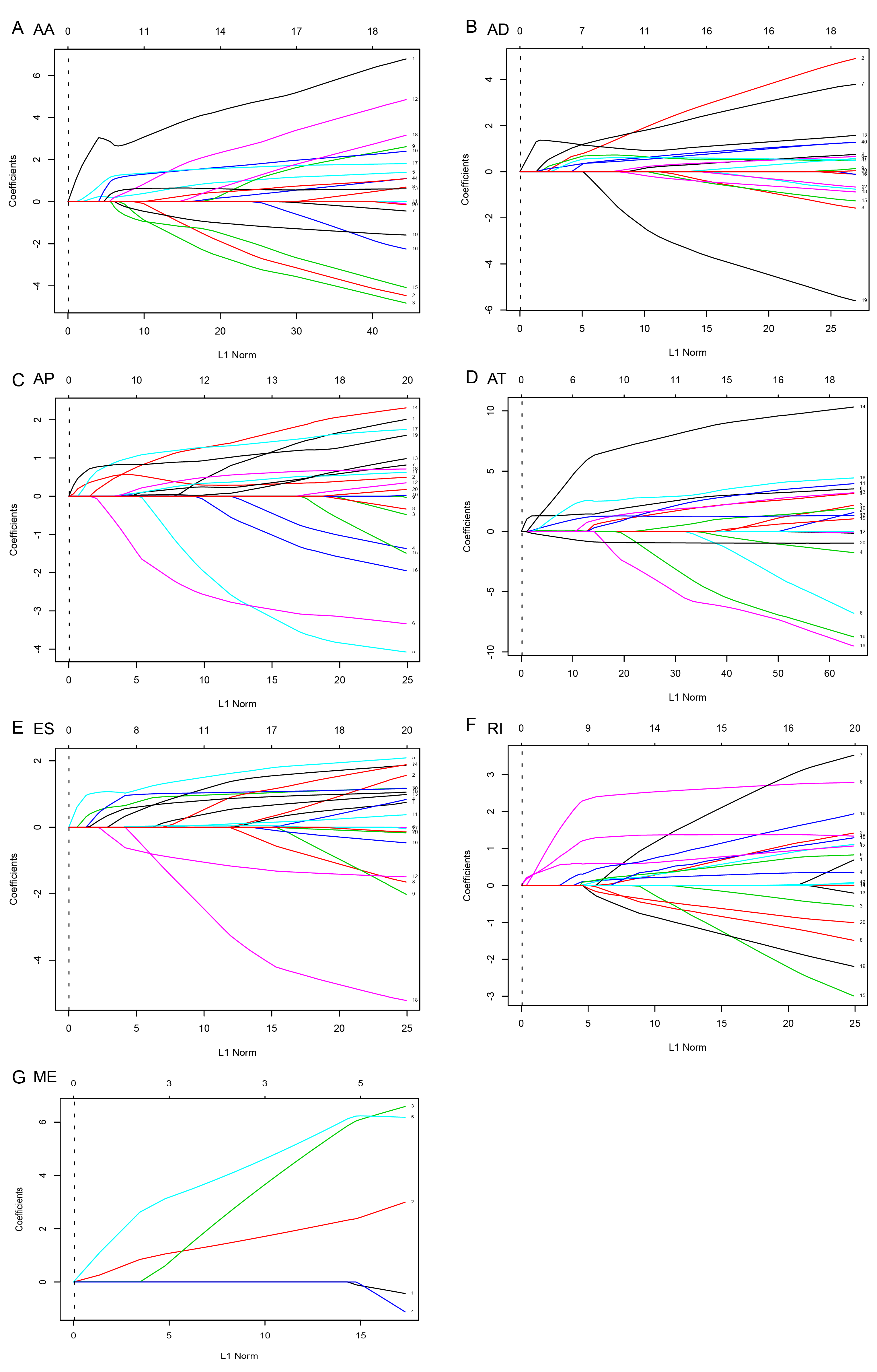

Supplement: Supplemental Material [file KBIE_A_1906096_SM0788.zip › Supplementary Figure S3.tif]

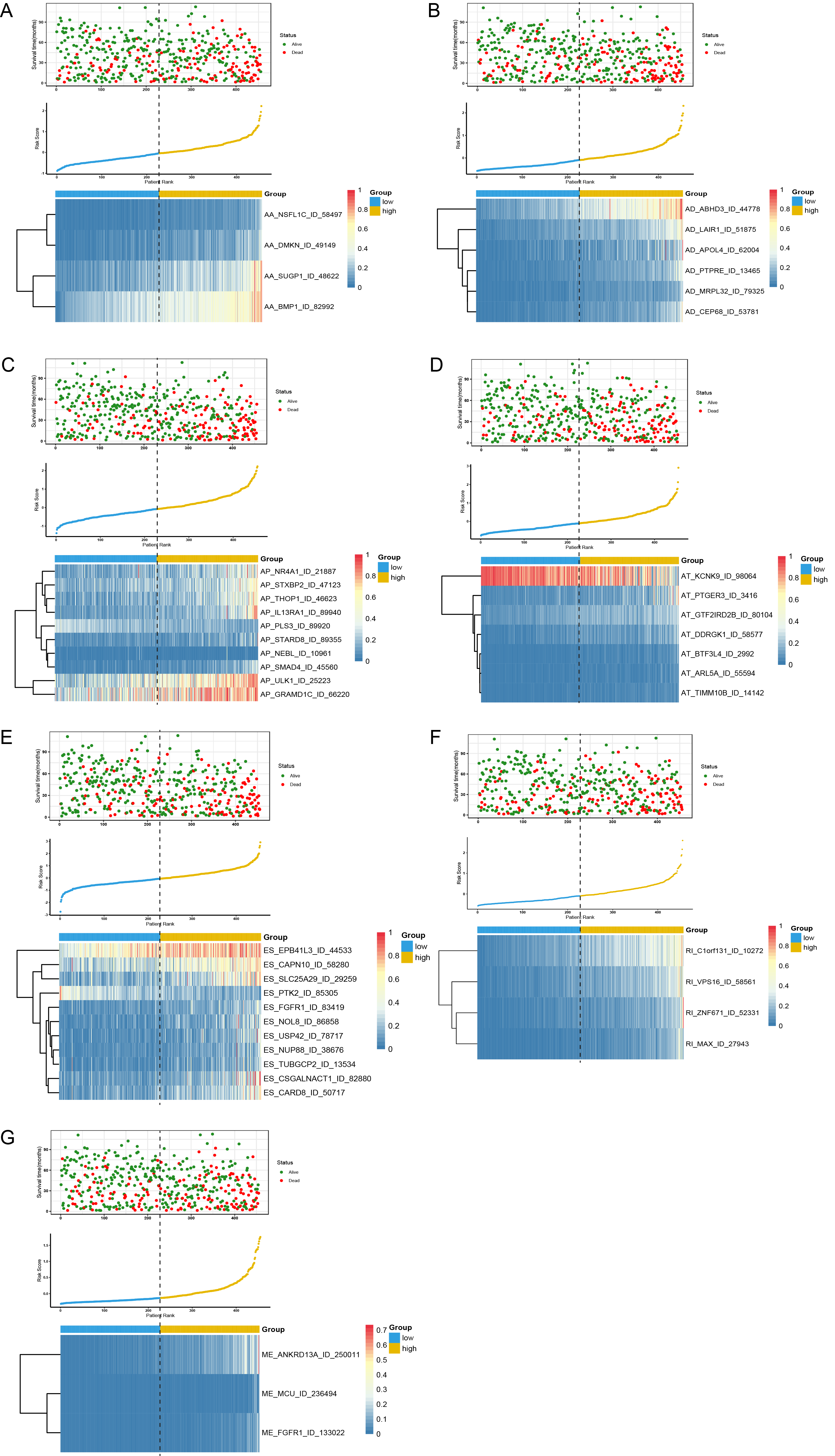

Supplement: Supplemental Material [file KBIE_A_1906096_SM0788.zip › Supplementary Figure S4.tif]

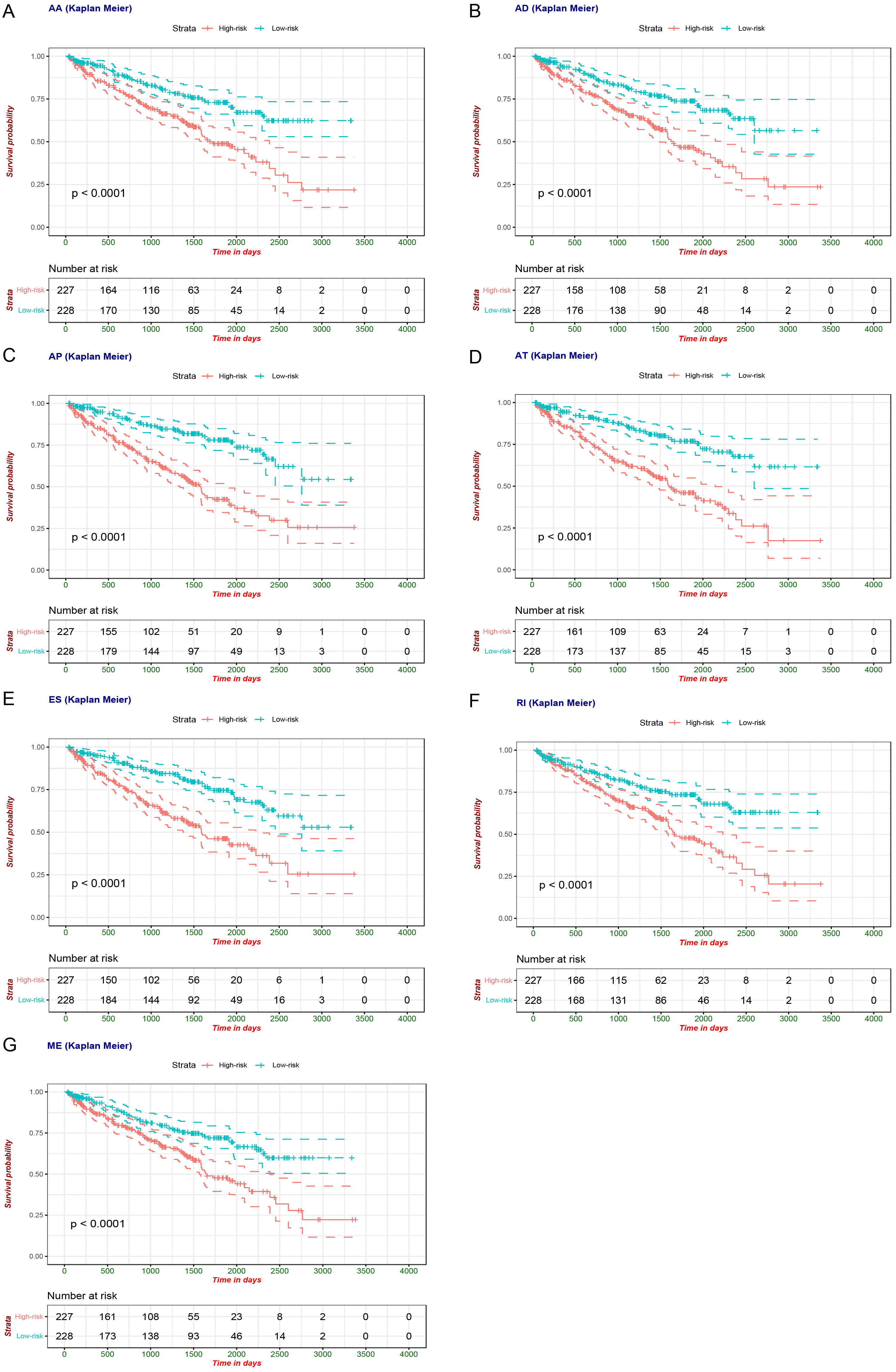

Supplement: Supplemental Material [file KBIE_A_1906096_SM0788.zip › Supplementary Figure S5.tif]

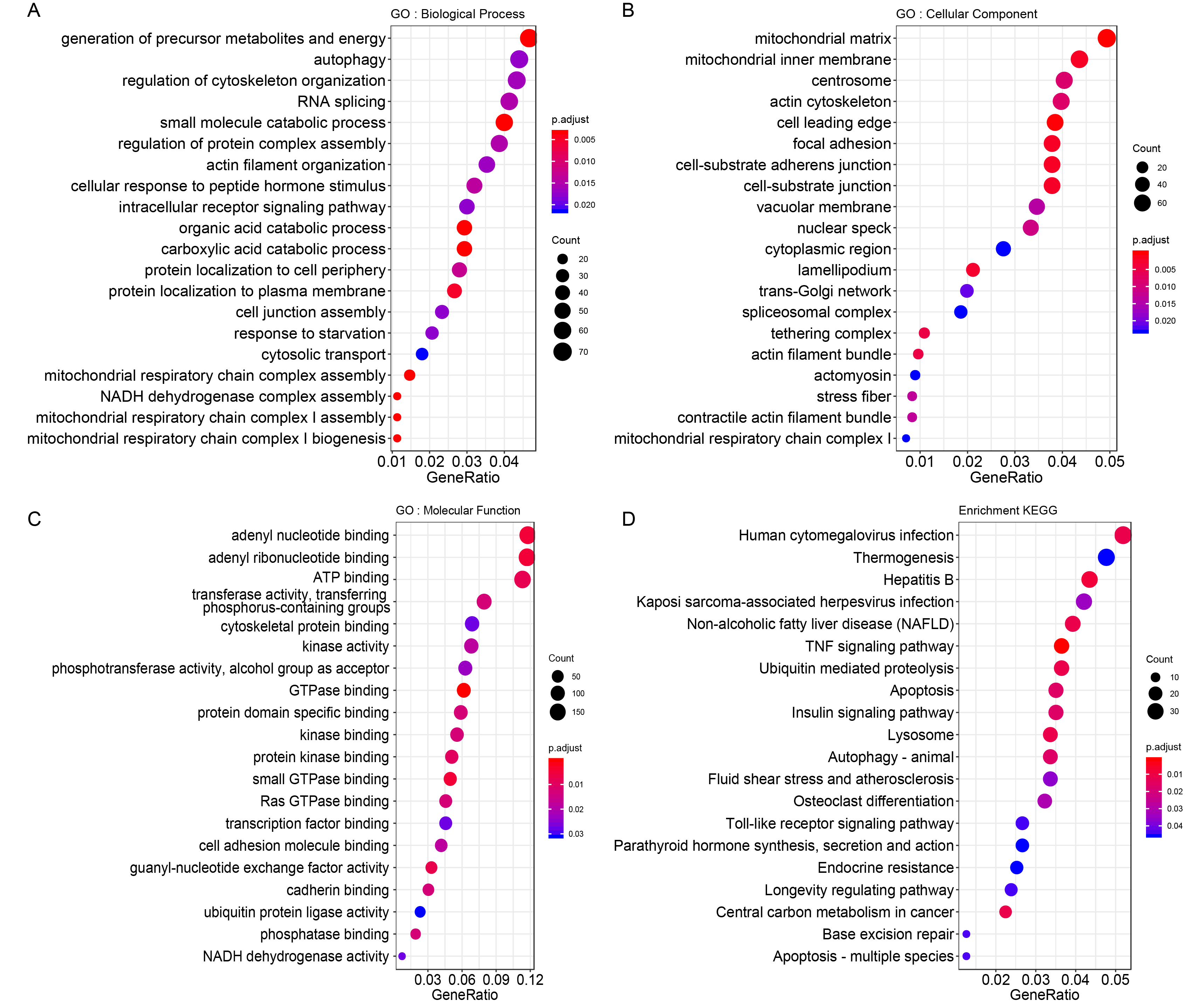

Supplement: Supplemental Material [file KBIE_A_1906096_SM0788.zip › Supplementary Figure S6.tif]

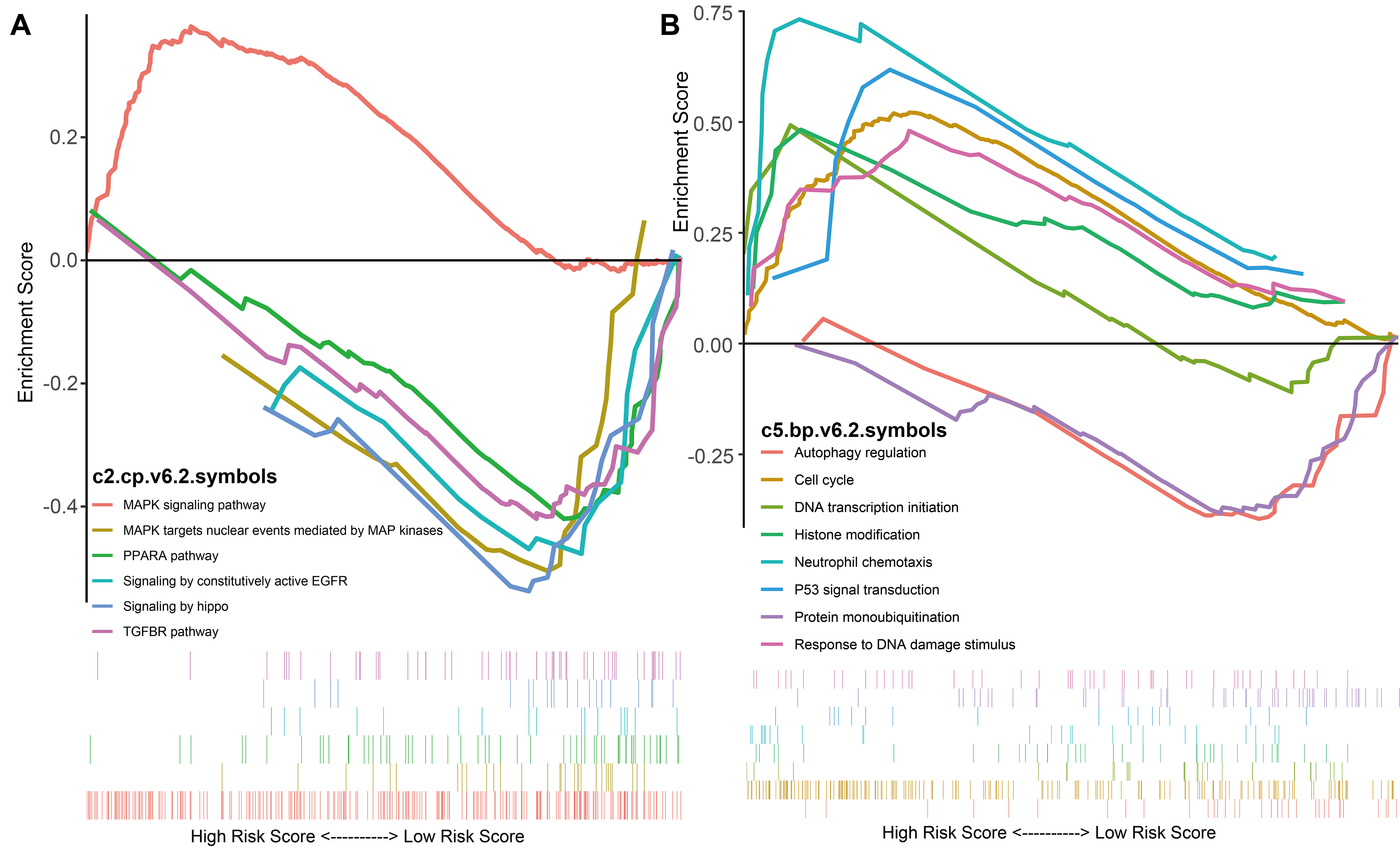

Supplement: Supplemental Material [file KBIE_A_1906096_SM0788.zip › Supplementary Figure S7.tif]

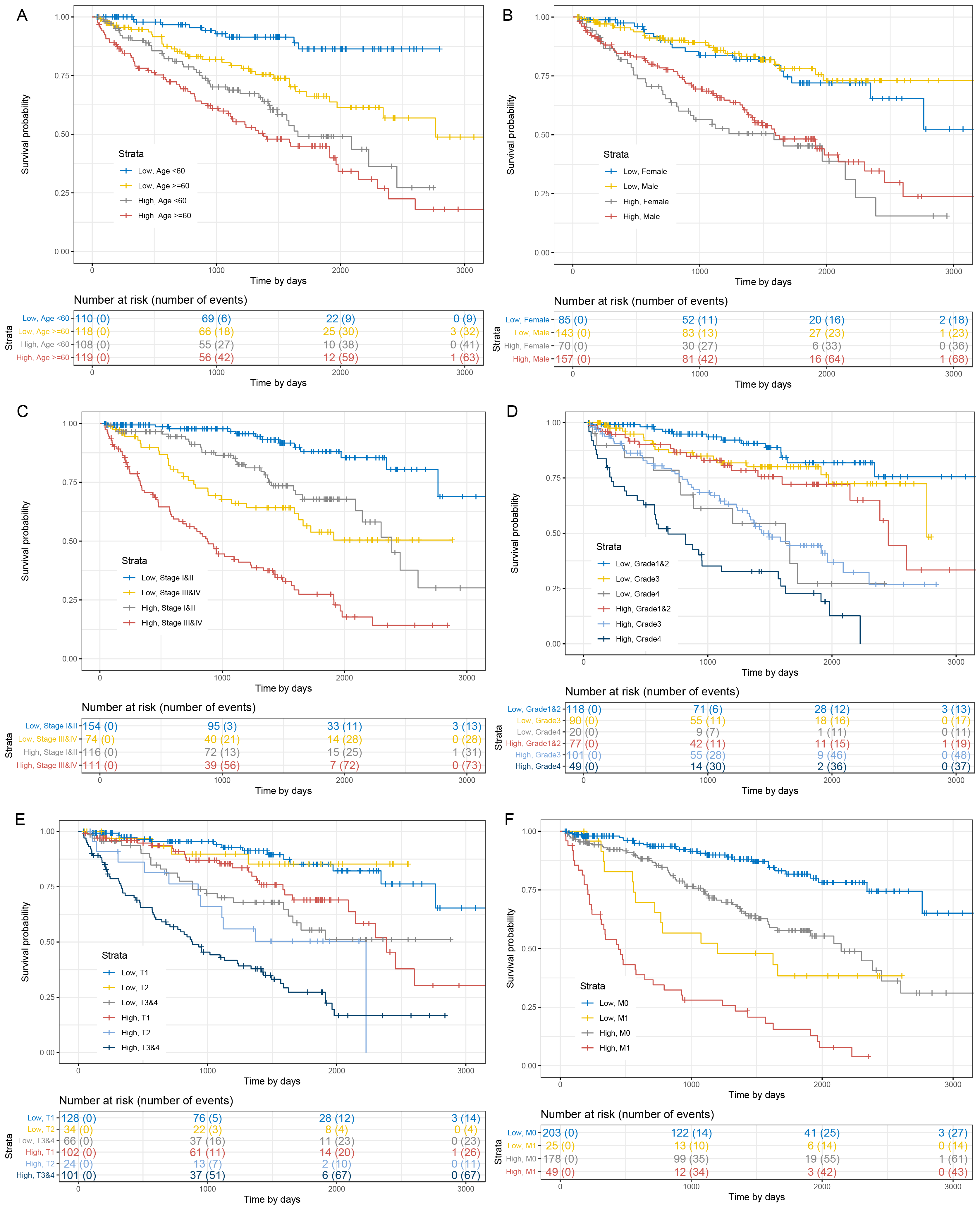

Supplement: Supplemental Material [file KBIE_A_1906096_SM0788.zip › Supplementary Figure S8.tif]

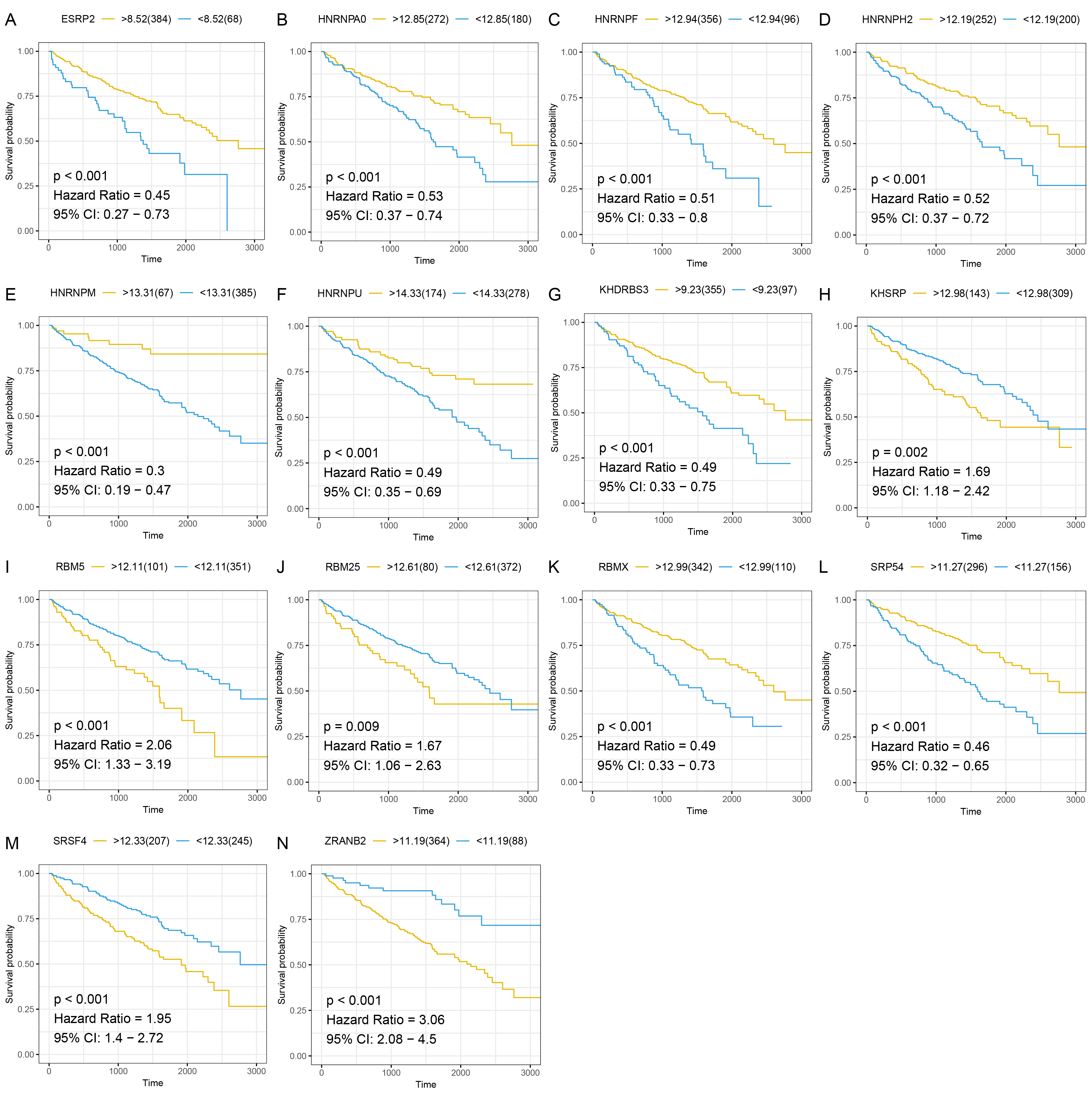

Supplement: Supplemental Material [file KBIE_A_1906096_SM0788.zip › Supplementary Figure S9.tif]
