# Supplementary material for: Novel insights into clear cell renal cell carcinoma prognosis by comprehensive characterization of aberrant alternative splicing signature: a study based on large-scale sequencing data
Source: Bioengineered. 2021 Mar 30;12(1):1091–110. doi: 10.1080/21655979.2021.1906096 (PMC8806224; doi:10.1080/21655979.2021.1906096)
Supplement: Supplemental Material [file KBIE_A_1906096_SM0788.zip › Supplementary Table S1.doc]

| **Supplementary Table S1：The detailed formula of each AS-based signature in ccRCC** | |
| --- | --- |
|  |  |
| **Signature Type** | **Formula** |
| AD | AD_MRPL32_ID_79325 * 1.176 + AD_PTPRE_ID_13465 * 0.831 + AD_CEP68_ID_53781 * 1.398 + AD_ABHD3_ID_44778 * 0.491 + AD_APOL4_ID_62004 * 0.770 + AD_LAIR1_ID_51875 * 1.093 |
| AP | AP_STXBP2_ID_47123 * 0.035 + AP_NEBL_ID_10961 * (-3.944) + AP_PLS3_ID_89920 * (-3.484) + AP_ULK1_ID_25223 * 0.399 + AP_NR4A1_ID_21887 * 0.519 + AP_THOP1_ID_46623 * 0.061 + AP_SMAD4_ID_45560 * 1.717 + AP_STARD8_ID_89355 * 1.355 + AP_GRAMD1C_ID_66220 * 0.741 + AP_IL13RA1_ID_89940 * 1.006 |
| ME | ME_ANKRD13A_ID_250011 * 2.581 + ME_MCU_ID_236494 * 6.698 + ME_FGFR1_ID_133022 * 6.791 |
| AT | AT_GTF2IRD2B_ID_80104 * 1.566 - AT_DDRGK1_ID_58577 * 0.169 + AT_ARL5A_ID_55594 * 1.943 + AT_TIMM10B_ID_14142 * 7.618 + AT_PTGER3_ID_3416 * 1.448 + AT_BTF3L4_ID_2992 * 3.068 + AT_KCNK9_ID_98064 * (-1.014) |
| AA | AA_NSFL1C_ID_58497 * 2.745 + AA_SUGP1_ID_48622 * 0.254 + AA_DMKN_ID_49149 * 1.787 + AA_BMP1_ID_82992 * 1.663 |
| RI | RI_ZNF671_ID_52331 * 2.557 + RI_VPS16_ID_58561 * 0.625 + RI_MAX_ID_27943 * 0.412 + RI_C1orf131_ID_10272 * 1.416 |
| ES | ES_CSGALNACT1_ID_82880 * 1.131 + ES_CARD8_ID_50717 * 1.949 + ES_FGFR1_ID_83419 * 1.978 + ES_EPB41L3_ID_44533 * 1.114 + ES_NOL8_ID_86858 * 0.239 + ES_PTK2_ID_85305 * (-1.482) + ES_USP42_ID_78717 * 0.985 + ES_NUP88_ID_38676 * 1.927 + ES_CAPN10_ID_58280 * 0.034 + ES_TUBGCP2_ID_13534 * (-5.632) + ES_SLC25A29_ID_29259 * 1.029 |
| Final | AD_PTPRE_ID_13465 * 0.508 + AD_CEP68_ID_53781 * 0.368 + AD_LAIR1_ID_51875 * 0.394 + AP_STXBP2_ID_47123 * (-0.508) + AP_PLS3_ID_89920 * (-3.244) + AP_STARD8_ID_89355 * 1.062 + AP_IL13RA1_ID_89940 * 0.239 + AT_TIMM10B_ID_14142 * 4.483 + AT_PTGER3_ID_3416 * 0.781 + AT_KCNK9_ID_98064 * (-0.306) + AA_DMKN_ID_49149 * 0.745 + AA_BMP1_ID_82992 * 0.390 + RI_ZNF671_ID_52331 * 0.744 + ES_CARD8_ID_50717 * 0.334 + ES_EPB41L3_ID_44533 * 0.986 + ES_PTK2_ID_85305 * (-1.282) + ES_SLC25A29_ID_29259 * 0.540 |
|  |  |
